# A novel insight into differential expression profiles of sporadic cerebral cavernous malformation patients with different symptoms

**DOI:** 10.1038/s41598-021-98647-9

**Published:** 2021-09-29

**Authors:** Hilal Eren Gozel, Kıvanç Kök, Fatma Ozlen, Cihan Isler, Sadrettin Pence

**Affiliations:** 1 Aziz Sancar Institute of Experimental Medicine, Department of Molecular Medicine, Istanbul University, Istanbul, Turkey; 2 Regenerative and Restorative Medicine Research Center (REMER), Health Sciences and Technology Research Institute (SABITA), Istanbul Medipol University, Istanbul, Turkey; 3 Department of Biostatistics and Medical Informatics, International School of Medicine, Istanbul Medipol University, Istanbul, Turkey; 4 Department of Neurosurgery, Cerrahpasa Medical Faculty, Istanbul University-Cerrahpasa, Istanbul, Turkey; 5 Department of Physiology, Faculty of Medicine, Istanbul Medeniyet University, Istanbul, Turkey

**Keywords:** Mechanisms of disease, Molecular medicine

## Abstract

Cerebral cavernous malformation (CCM) is a vascular lesion of the central nervous system that may lead to distinct symptoms among patients including cerebral hemorrhages, epileptic seizures, focal neurologic deficits, and/or headaches. Disease-related mutations were identified previously in one of the three CCM genes: *CCM1*, *CCM2,* and *CCM3*. However, the rate of these mutations in sporadic cases is relatively low, and new studies report that mutations in CCM genes may not be sufficient to initiate the lesions. Despite the growing body of research on CCM, the underlying molecular mechanism has remained largely elusive. In order to provide a novel insight considering the specific manifested symptoms, CCM patients were classified into two groups (as Epilepsy and Hemorrhage). Since the studied patients experience various symptoms, we hypothesized that the underlying cause for the disease may also differ between those groups. To this end, the respective transcriptomes were compared to the transcriptomes of the control brain tissues and among each other. This resulted into the identification of the differentially expressed coding genes and the delineation of the corresponding differential expression profile for each comparison. Notably, some of those differentially expressed genes were previously implicated in epilepsy, cell structure formation, and cell metabolism. However, no *CCM1-3* gene deregulation was detected. Interestingly, we observed that when compared to the normal controls, the expression of some identified genes was only significantly altered either in Epilepsy (*EGLN1*, *ELAVL4*, and *NFE2l2*) or Hemorrhage (*USP22*, *EYA1, SIX1*, *OAS3*, *SRMS*) groups. To the best of our knowledge, this is the first such effort focusing on CCM patients with epileptic and hemorrhagic symptoms with the purpose of uncovering the potential CCM-related genes. It is also the first report that presents a gene expression dataset on Turkish CCM patients. The results suggest that the new candidate genes should be explored to further elucidate the CCM pathology. Overall, this work constitutes a step towards the identification of novel potential genetic targets for the development of possible future therapies.

## Introduction

Cerebral Cavernous Malformation (CCM) is a disease characterized by the formation of a single or multiple lumen formation in continuous capillaries in the central nervous system which may result into the leakage of the veins or defects in the blood–brain barrier^1^. CCM may cause serious neurological symptoms including epileptic seizures (40–70%), hemorrhagic stroke (30–40%), and headache (10–30%)^2^. The prevalence of CCM is 0.1–0.5% in the general population. It was thought that there are 18 to 22 million CCM patients worldwide^3^.

CCM has both sporadic (80%) and familial (20%) forms^4^. Approximately within 50% of familial and sporadic cases, loss-of-function mutations are observed in at least one of the three genes: *CCM1* (K-Rev interaction trapped 1, KRIT1), *CCM2* (OSM or malcavernin) and *CCM3* (programmed cell death 10, PDCD10)^5^. In familial cases, the frequencies of *CCM1*, *CCM2* and *CCM3* mutations were approximately 53–65%, 15–19%, and 10–22%, respectively^6–8^. Notably, in the remaining %50 of all cases, no mutation is detected in any CCM genes. Thus, it could be that some genes related to this disease remain elusive. Relatedly, 16 years have passed since the identification of *CCM3* gene^9^, yet no more CCM-related genes were discovered. This emphasizes the need for a novel experimental approach to fill in this research gap.

CCM is thought to be associated with endothelial cells and surrounding glial cells, which could be a possible explanation for why lesions are majorly localized in the brain and spinal cord^10^. Although the exact functions of the three *CCM* genes are not known precisely, some studies demonstrated that the corresponding encoded proteins may alter the integrity of endothelial permeability by inhibiting RhoA-associated kinase (ROCK) activation^11,12^. This switches on a transcription factor named bone morphogenic protein (BMP) which triggers the activation of transforming growth factor-beta (TGF-beta) and may cause an endothelial-to-mesenchymal transition (EndoMT)^13^. Furthermore, these genes have a role in several biological processes including inflammation, apoptosis, cell polarity, angiogenesis, cellular adhesion, cytoskeletal reorganization, endothelial stress response, and the regulation of blood vessel architecture, as well as, in some signaling pathways such as MEKK3-KLF2/4, TGF-β/BMP, Wnt/β-catenin and Notch pathways^10,14^.

The following question remained unanswered until now: Why some patients without any mutations at the three *CCM* loci still develop CCM lesions? Moreover, the prevalence of having mutations in one of *CCM1-3* genes varies in different populations. Verlaan et al*.* found no *CCM2* mutation in 31 German patients^15^. Another study with 40 Italian patients reported relatively low detected mutations in CCM genes unlike the French, Swiss and Japanese cohorts. They observed *CCM1* mutation only in two patients and *CCM2* in a single patient. The remaining 92.5% patients had no mutation in CCM genes^16^.

New evidence put forward that the mutations in these three CCM genes alone may not be sufficient to initiate CCM lesions which suggests that there may be some unidentified possible genetic factors for the disease pathology^17,18^.

Expression-based approach or transcriptome analysis provides an essential tool for profiling genome-wide differential RNA expression to obtain alternative insights for disease progression.

To detect the novel genes, functions, and networks contributing to the pathobiology of CCM, Koskimäki et al. performed transcriptomic analysis across 3 species (human, mouse and *Caenorhabditis elegans*) and 2 disease genotypes (induced loss of *CCM1* and *CCM2* genes) from laser microdissected neurovascular units. They revealed common genes and 1 GO (Gene Ontology) term (GO:0051656, establishment of organelle localization) along with 24 GO functions that were present in 4 of 5 organisms. Those GO functions were associated with cell-to-cell adhesion, neutrophil-mediated immunity, ion transmembrane transporter activity, and responses to oxidative stress^19^.

In 2019, Lyne et al*.* suggested a fresh symptom-based-approach by comparing Cavernous Angiomas with Symptomatic Hemorrhage (CASH) with healthy individuals and Non-Cash patients^20^. They identified some shared and unique biomarkers of CASH with lesional transcriptome analysis.

A recent article that executed transcriptomic analysis on endothelial cells isolated from two sporadic CCM specimens found no mutations on *CCM1-3* genes. Remarkably, they suggested novel candidate genes, and a possible explanation for disease pathology that may be due to a molecular shift from canonical to non-canonical Wnt pathway^21^. Another article investigated the transcriptomic similarities in the aging brain and CCM samples to explore common pathological mechanisms predisposing patients to hemorrhage^22^.

**Figure 1:**
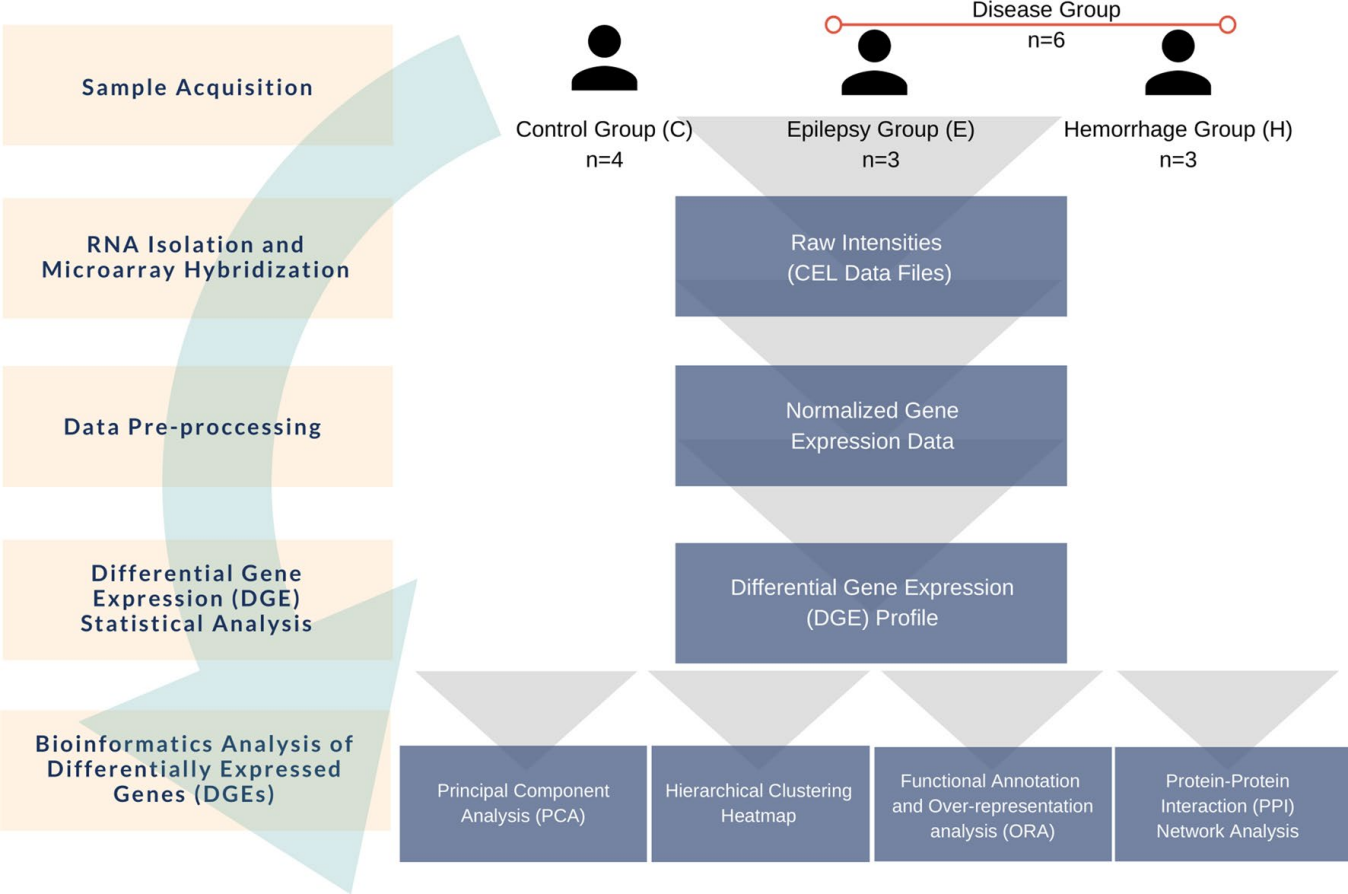
Experimental design.

In summary, these studies suggest that novel approaches and brand-new perspectives are required to elucidate the complex pathology of CCM, its disease-related molecules, interactions of these molecules and signaling pathways that contain these molecules. In the presented study, we hypothesize that the different groups of proteins are involved in the pathogenesis of sporadic CCMs with different symptoms. Therefore, we focused on coding genes, which were differentially expressed between the studied groups. In this regard, we report results of the first comparative transcriptomic data analysis of Turkish CCM patients and the control group. The patients were divided with this purpose into two groups based on the different symptoms, namely epilepsy and hemorrhage, with sporadic CCM (Fig. 1).

## Results

Cerebral cavernous malformation (CCM) is a vascular formation with various symptoms among patients including cerebral hemorrhages, epileptic seizures, focal neurologic deficits, and/or headaches. With transcriptome analysis of the patients with distinct symptoms, it was aimed to compare the differentially expressed genes in patients with Epilepsy or Hemorrhage. The experimental (wet lab) part of this study consisted of Sample Retrieval. RNA Isolation and Microarray Analysis Screening. This was followed by pre-processing of the generated raw gene expression data. The resulting normalized gene expression data, corresponding to the total of 53,617 probesets, was used as input for differential expression analysis.

### Differential expression analysis

**Figure 2:**
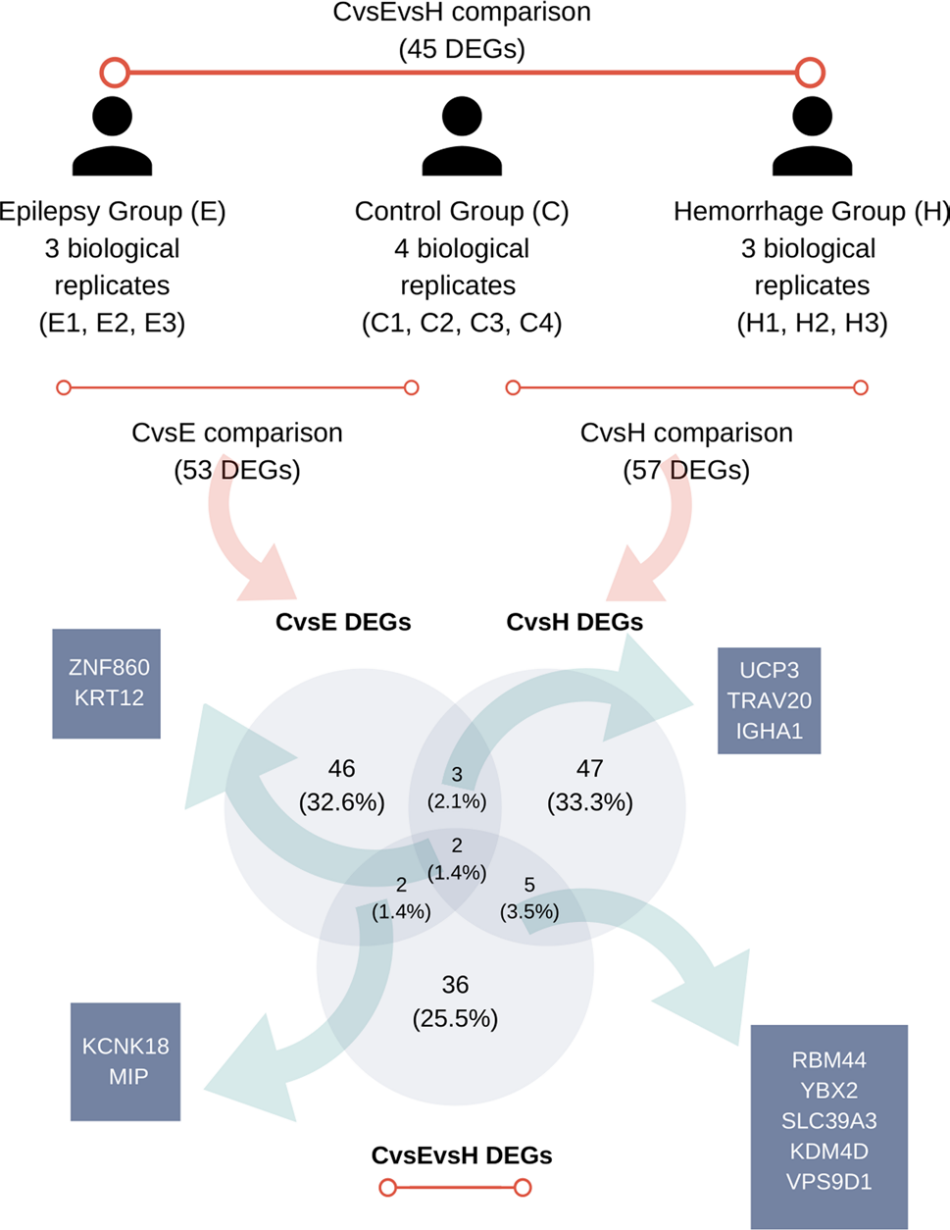
Description of sample groups (E, H, and C) and Venn diagram for common genes between CvsE (control vs epilepsy), CvsH (control vs hemorrhage), and CvsEvsH (control vs epilepsy vs hemorrhage) DEG (differentially expressed gene) lists.

For differential expression analysis (DEA), three different comparisons were performed. In the CvsE DEA analysis, control samples were compared to the samples from patients having epilepsy; in the CvsH DEA analysis, control samples were compared to the samples from patients having hemorrhage; in the CvsEvsH DEA analysis, control samples and patients with epilepsy and hemorrhage were compared altogether. Consequently, among the significant probesets (*p* < 0.05), the top 200 probesets (200 probesets with the lowest p values) were identified for each of three comparisons. In the subsequent analysis, only probesets for protein-coding genes were included (all other probesets were excluded). Prior to further analysis, these probesets were converted into HGNC gene symbols, and constituted the corresponding differentially expressed genes (DEG) lists. More specifically, the remaining probesets corresponded to 53 DEGs for the CvsE comparison (Supp. Table S2), 57 DEGs for the CvsH comparison (Supp. Table S3), and 45 DEGs for the CvsEvsH group comparison, respectively (Supp. Table S4). Remarkably, the delineated differential expression profiles highlighted reduced expression of all DEGs in the disease samples, when compared to controls. Figure 2 shows the Venn diagram of common genes between the CvsE, CvsH and CvsEvsH comparisons results. As a result of this survey, two genes present in all 3 lists were identified (ZNF860 and KRT12). Five genes shared between the CvsE and CvsH groups were identified (UCP3, TRAV20, IGHA1, ZNF860 and KRT12). Four genes common between the CvsE and CvsEvsH lists were identified (KCNK18, MIP, ZNF860 and KRT12). Seven genes common between the CvsH and CvsEvsH lists were identified (RBM44, YBX2, SLC39A3, KDM4D, VPS9D1, ZNF860 and KRT12).

### Principal component analysis

**Figure 3:**
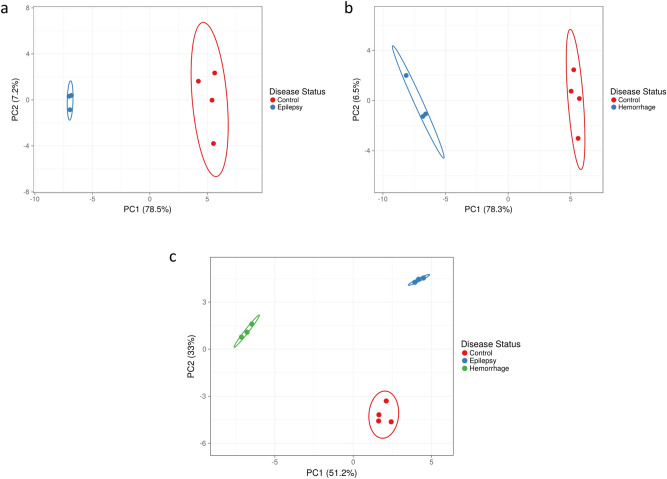
Principal component analysis (PCA) of the analyzed samples separates samples by sample origin based on the expression profile of differentially expressed genes (DEGs). PCA plots were obtained for the CvsE (**a**), CvsH (**b**), and CvsEvsH (**c**) comparisons, respectively, based on the expression profile of the corresponding differentially expressed genes (DEGs). 95% prediction ellipses indicate that a new sample obtained from the same groups, are expected to be localized in the depicted ellipse area with a probability 0.95. The PC1 (principal component 1) and PC2 (principal component 12) or the CvsE comparison corresponded to an explained variance of 78.5% and 7.2%, respectively. This indicates that new observations with the same groups are expected to coincide with similar regions with a probability of 0.95 (**a**). For the CvsH comparison, the variances of represented by the PC1 and the PC2 were obtained as 78.3% and 6.5%, respectively. New observations to be made with the same sample groups is expected to coincide with similar regions with a probability of 0.95 (**b**). For the CvsEvsH comparison, the variances of covered by the PC1 and PC2 were found as 51.2% and 33%, respectively. New observations to be made with the same groups will is expected to coincide with similar regions with a probability of 0.95 (**c**).

Principal component analysis (PCA) analysis, which was based on the gene expression data of the identified DEGs, was performed separately for each DEG list. Obtained PCA plots revealed grouping of the analyzed samples according to their sample group. In another words, the intragroup variation was less than intergroup variation. The sum of the variation captured by the first and the second Principal Component was high enough to make reliable conclusions about the observation (Fig. 3).

### Hierarchical clustering analysis

**Figure 4:**
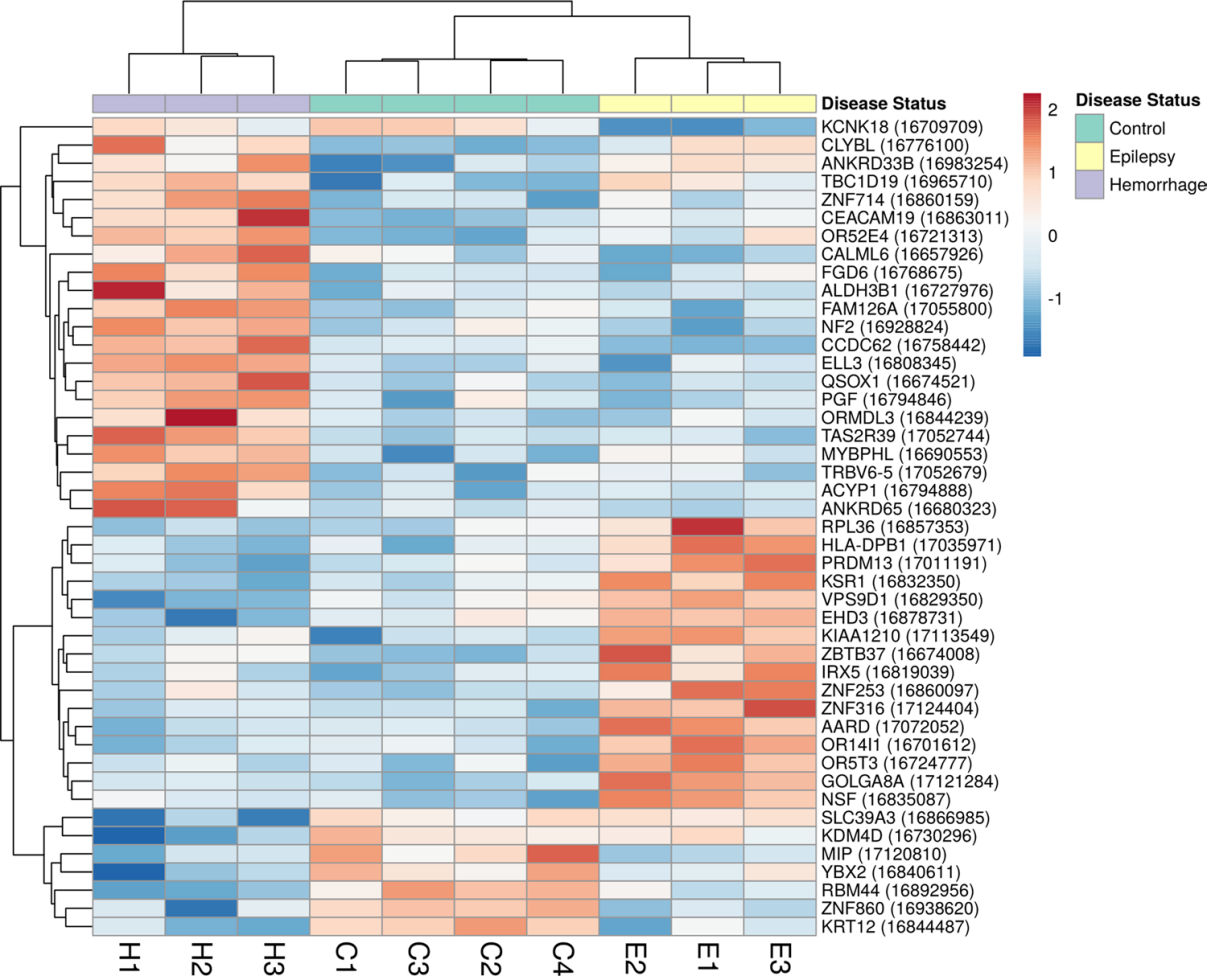
Hierarchical clustering heatmap for the expression of CvsEvsH DEGs. The delineated differential gene expression DGE profile highlights clustering of samples according to their sample group. Red color and blue color show relatively higher and lower gene expression, respectively.

Hierarchical clustering heatmap plots were generated for the obtained DEG lists using the hierarchical clustering analysis. The sample dendrogram in each of the three different heatmap plots demonstrated grouping of samples according to their sample group [CvsE (Supp. Fig. S1), CvsH (Supp. Fig. S2) and CvsEvsH (Fig. 4)]. These results are in line with the obtained PCA results.

Altogether, the robust separation pattern in the PCA and hierarchical clustering plots suggest indicative value of the identified DEGs for the discrimination between the study groups.

### Functional annotation analysis

The functional annotation was performed separately for the 3 DEG lists using a GO Slim summary and Over-representation analysis (ORA). The ORA was performed to identify enriched functional annotation terms using four independent databases, namely KEGG (Supp. Fig. S3), Wikipathway (Supp. Fig. S4), Panther (Supp. Fig. S5), and Reactome (Supp. Fig. S6).

Evaluation of the obtained CvsE DEGs-related ORA results highlighted, among others, the following functional annotation categories: biological regulation, metabolic process, and cellular component organization. Interestingly, CvsH DEGs were related, among others, with the following functional annotation categories: in biological regulation, metabolic processes, and multicellular organismal process. Most of CvH DEGs were shown to be located on membrane.

**Figure 5:**
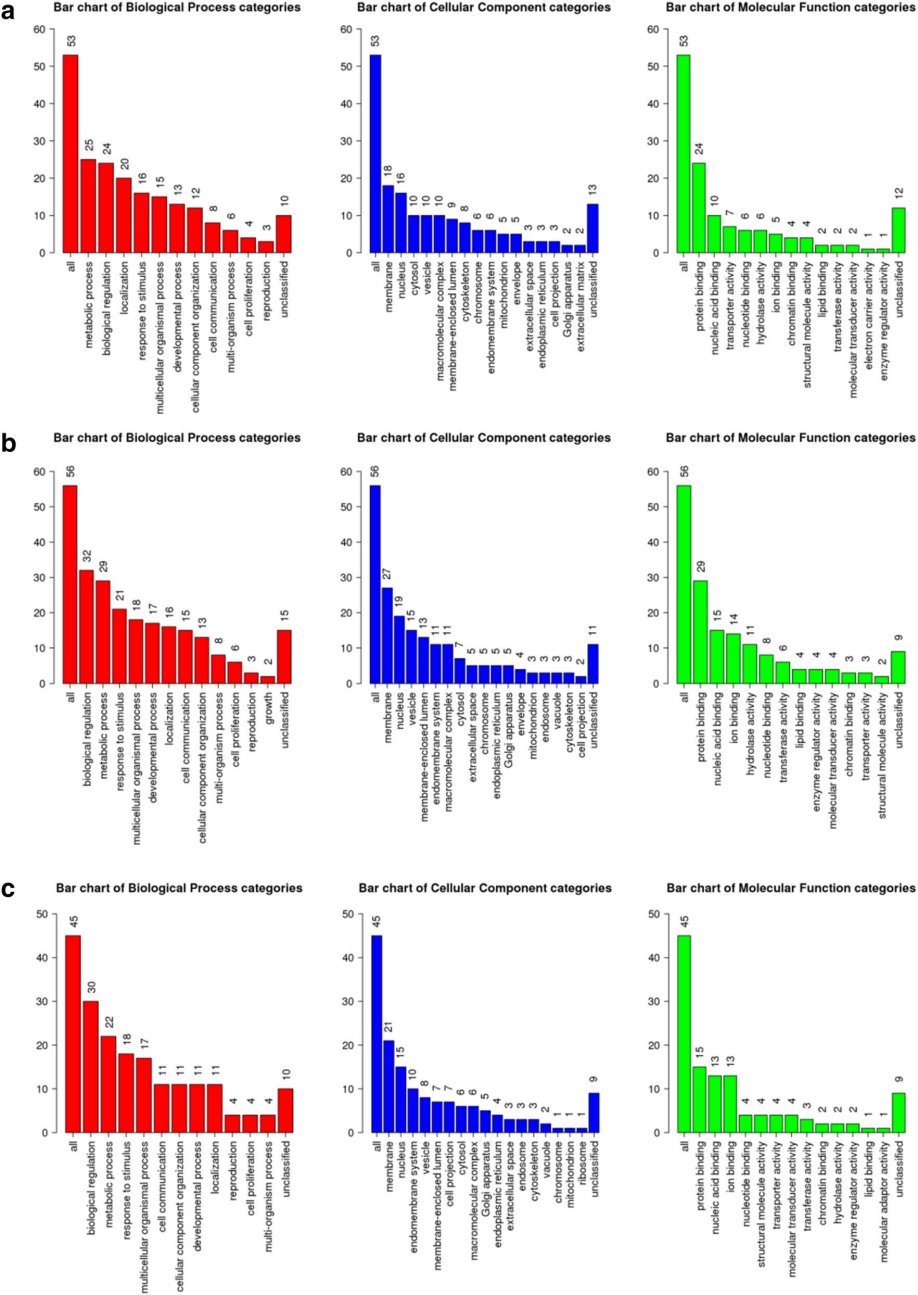
Results of GO slim summary for CvsE (**a**), CvsH (**b**), and CvsEvsH (**c**) DEGs, respectively. As a standard practice for this cut-down GO representation, the input genes were directly mapped to the standard GO subset based on the underlying GO annotation. The Y axis shows number of genes associated with each presented representative GO category. This plot is not based on statistical significance but solely on distribution of genes in the set of pre-fixed categories, selected by the GO database experts.

Finally, functional annotation of the CvsEvsH DEGs emphasize a role in processes, such as biological regulation, metabolic processes, and response to stimuli. Notably, protein products of the explored DEGs are mostly located on the membrane (Fig. 5).

As the GO Molecular Functions, associated with the investigated genes, are examined, the two top categories, in terms of frequency, are ‘protein binding’ and ‘nucleic acid binding’. When other top categories are examined, some differences between the DEG lists become apparent. While results for the CvsE DEG list contain functions as ‘transporter activity’, ‘nucleotide binding’, and ‘hydrolase activity’. The CvsH DEGs-related results included functions such as ‘ion binding’, ‘hydrolase activity’, and ‘nucleotide binding’. Most of the proteins encoded by the genes in the CvsEvsH list have functions such as ‘ion binding’, ‘nucleotide binding’, and ‘participation in the structure’. Overall, the performed functional annotation provided an overview of the features and functions, known to be associated with the identified DEG lists.

### Protein–protein interaction network analysis

**Figure 6:**
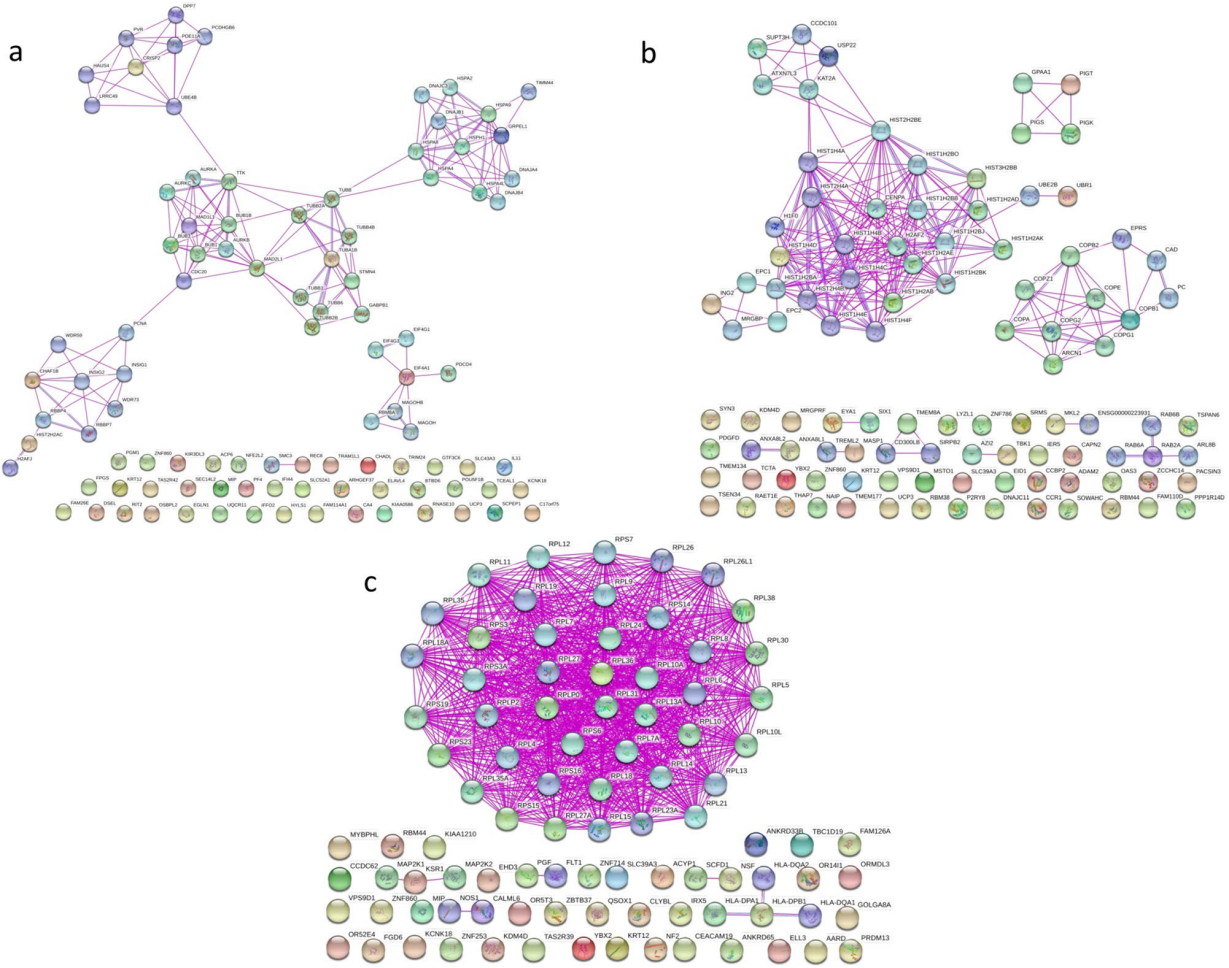
Protein–protein interaction network (PPI) analysis for the CvsE (**a**), CvsH (**b**), and CvsEvsH (**c**) DEG lists. Nodes represent proteins and edges represent protein–protein interactions. The main interaction map consists of seed proteins protein (encoded by the DEGs) and their direct interaction partners (first degree interactors identified by the String database). The DEGs without any connection are listed at the bottom of the figure (More detailed version is available in Supplementary as Figs. S7–S9, respectively).

Protein–Protein Interaction Network Analysis (PPI) was performed in order to explore interactions of the DEGs by reconstructing the underlying interaction networks (separately for each gene list). As a result, PPI maps were created for the CvsE (Fig. 6a), CvsH (Fig. 6b) and CvsEvsH DEG lists (Fig. 6c) (More detailed version is available in Supplementary as Figs. S7–S9, respectively). The network consisted of the mapped input genes and their direct (first degree) interaction partners. The resulting networks comprised 98, 103 and 92 gene products (represented as nodes) for the input CvsE, CvsH and CvsEvsH DEGs. The PPI network revealed known interactions among the studied genes and their direct interaction partners.

## Materials and methods

### Sample retrieval

In this study, six paraffin embedded cavernous tissue specimens from CCM patients without known clinically affected relatives, and four paraffin embedded control brain tissue specimens were used following the ethical approval. Sporadic CCM patient samples were obtained from the patients that were diagnosed and underwent surgery in the Department of Neurosurgery at Istanbul University-Cerrahpasa. Following formalin fixation, these samples were embedded in paraffin. 6 samples were categorized equally as Epilepsy (n = 3) and Hemorrhage (n = 3) groups in accordance with their symptoms. Four paraffin embedded control tissues were provided by the Istanbul Forensic Medicine Institute, after the necessary permits were obtained. These tissues have been fixed in formalin and then embedded in paraffin in Istanbul Forensic Medicine Institute. Details of samples are present in Table S1 in the Supplementary part. The experimental design is presented in Fig. 1. All procedures performed in studies involving human tissues were in accordance with the ethical standards of the institutional and/or national research committee and with the 1964 Helsinki Declaration and its later amendments or comparable ethical standards and informed consent was obtained from all participants and/or their legal guardians. This study was approved by the Bioethics Committee of the Faculty of Medicine, Istanbul University (Number: 960).

### RNA isolation

Prior to the experiment, RNase inhibitor (RNAase ZAP, Sigma) was applied to the equipment. Tissues were incubated with xylene and ethanol baths 55 °C to remove paraffin and powdered with liquid nitrogen in ceramic mortar. 100 µg of powdered tissues were incubated with trizol agent (TriReagent, Sigma) on ice for 10 min, vortexed vigorously, and centrifugated at 12,000×*g* for 15 min to remove cell debris. Then 0.2 ml ice-cold chloroform was added for extraction. Samples were vortexed, incubated for 5 min on ice and centrifugated at 12,000×*g* for 15 min at 4 °C. Upper aqueous phase was transferred to new tubes carefully and mixed with equal volume of isopropyl alcohol for precipitation. Samples were incubated at 20 °C for 30 min and centrifugated at 12,000×*g* for 10 min at 4 °C. For RNA wash, supernatant was removed completely, and pellet was incubated with ice-cold 70% ethanol for 10 min at 4 °C. Samples were centrifugated at 7500×*g* for 5 min at 4 °C. This washing step was repeated twice. Following second centrifugation, ethanol was removed carefully, and the pellet was air-dried. Finally, 20 μl RNase free water was added for elution of RNA. RNA concentration and purity was measured with a NanoDrop instrument (P360 Implen nanophotometer, Germany), and only RNA with OD 260/280 ≥ 1.8 was selected for further analysis. Equal quantities of RNA from each tissue sample were used for cDNA synthesis and sequencing.

### Microarray analysis screening

SensationPlus™ FFPE Amplification and WT Labeling (Affymetrix, Thermo Fisher Scientific, USA) kit was used to perform the whole genome transcription expression analysis. With this kit, reverse transcription from total RNA was used to produce cDNA, and then cDNA was amplified and increased in quantity. After the amplified cDNAs were purified according to the kit; fragmentation, terminal end labeling and array chip hybridization were performed, respectively. Throughout the screening experiment, Human Gene 2.0 ST (HuGene) (Affymetrix, USA) microarray chips were used. The experiment generated CEL data files, which contained probe-based raw intensity data.

### Data pre-processing analysis

The obtained CEL data files were pre-processed using R software environment^23^ and its Bioconductor Graphical User Interface (GUI) (version 3.5)^24^. The *Affy*^25^ and *limma*^26^ R packages (version 3.4.1, RRID:SCR_001320) were used to read in and analyze the Affymetrix-compatible files in this step. The probe-level data corresponding to the 53,617 probesets (for each sample) were normalized using the Robust Multi-array Average-(RMA) method^27^ and transformed into normalized gene expression data.

### Differential expression analysis

After pre-processing, analysis of differential expression was performed to identify differentially expressed coding genes (DEGs). As described above, the studied sample groups were defined according to the three different morphological characteristics of the disease (control, hemorrhage and epilepsy). In the CvsE comparison, controls (n = 3) were compared to patients having epilepsy (n = 3). Then in the CvsH comparison, controls (n = 3) were compared to patients having hemorrhage (n = 3). Finally, in CvsEvsH comparison, the multigroup comparison (namely, 3 groups comparison) was performed. The normalized dataset obtained as result of the data pre-processing analysis was used as input for the differential expression analysis (DEA). The aim of these comparisons was to distinguish probesets with differential expression pattern from the rest of the probesets in the normalized dataset. The statistical analysis was performed using Class comparison functionality of the Babelomics (version 5, accessed on December 2020, RRID:SCR_002969) (http://babelomics.bioinfo.cipf.es). *Limma* method was applied with the cut off value of *p* < 0.05 for the statistical testing of significance^26^. The output of each performed comparison consisted of a list of significant differentially probesets (*p* < 0.05). Herein, all significant probesets in each list were ordered according to their respective p values and the top 200 probesets (200 probesets with the lowest p value) were prioritized for further analysis.

In the next step, among these top probesets, only those corresponding to protein coding genes remained (all other probesets were excluded). Finally, the probeset IDs of the remaining probesets were converted into gene symbols assigned by the HUGO Gene Nomenclature Committee (HGNC, RRID:SCR_002827). The obtained list of coding genes constituted the DEG list for the respective comparison. Subsequently, Venn diagrams were generated with Venny (version 2.1.0, accessed on December 2020, RRID:SCR_016561) program (http://bioinfogp.cnb.csic.es/tools/venny/) to demonstrate the presence of common genes between the resulting DEG lists.

### Bioinformatics analysis of differentially expressed genes

Principal Component Analysis (PCA)^28^ and hierarchical clustering analysis was applied to assess differences between the sample groups based on the gene expressions of the corresponding DEGs. The PCA plots and hierarchical clustering heatmaps were generated using ClustVis program (https://biit.cs.ut.ee/clustvis/) (BETA version, accessed on December 2020, RRID:SCR_017133) separately for each DEG list. PCA analysis was performed using the following default methods: Unit variance scaling was applied to rows and singular value decomposition (SVD) was used to calculate principal components (PCs).

For the hierarchical clustering analysis, the following default methods were selected: Rows were centered, unit variance scaling was applied to rows, Pearson correlation was used as clustering distance and average linkage was applied as clustering method. Both samples and DEGs were subjected to hierarchical clustering in order to analyze and visualize the related differential gene expression (DGE) profiles.

The first step in Functional annotation was accomplished using the Gene Ontology (GO) Slim representations. GO Slim (http://geneontology.org/docs/go-subset-guide/) is a standardized overview annotation based on the cut-down of GO. Instead of any statistical inference, this approach simply provides a numerical summary of GO associations, based on the input gene list and the representative GO categories. This was followed by a complementary analysis which included four independent parts, each for a separate functional annotation resource, namely KEGG, Wikipathway, Panther, and Reactome databases (by choosing the “pathway” option). In the ORA analysis, the default “Top 10” option was selected. This resulted in identification of top categories (having the lowest p-value, obtained as result of enrichment analysis). As the next, significant categories (*p* < 0.05) among the top categories were highlighted. Functional annotation was performed via WebGestalt (WEB-based Gene SeT Analysis Toolkit) program (http://www.webgestalt.org/) (version/update 2013, accessed on December 2020, RRID:SCR_006786). The aim was to facilitate functional interpretation of the identified DEG lists as result of DEA. Consequently, the three DEG lists were used separately as input in this step to gain insights into potential functional consequences of the delineated DEG profiles. In the course of the functional annotation step, except for the GO Slim part, Over-representation Analysis (ORA) was performed.

String (version 10.5, accessed on December 2020, RRID:SCR_005223) program (https://string-db.org/) was used to retrieve the protein–protein interactions (PPI) of the studied proteins with their first-degree interaction partners to reconstruct the PPI networks in different groups. The network analysis was accomplished separately for each performed comparison by using the corresponding DEG list as input. For retrieving PPIs, minimum required interaction score was selected as ‘medium confidence:0.400’, the meaning of network edges was selected as ‘evidence’, active interaction source was selected as ‘experiments’, and the maximum number of interactors was selected as ‘1st shell – no more than 50 interactions’. In the program interface, the following configuration option was applied: ‘Multiple proteins’ → ‘Organisms (*Homo sapiens*)’ → ‘Settings’ → ‘Experiments’ → ‘1st shell no more than 50 interactions’.

## Discussion

Proteins, which are known to be associated with Cerebral Cavernous Malformations, particularly interfere with the formation of the vascular structure and rearrange the normal development process of this structure. Although many studies in the literature have shown that *CCM1-3* genes known to be associated with CCM may cause vascular dysfunction^5^, some studies suggest that there may be alternative genes associated with CCM, and *CCM1-3* genes alone are not sufficient to cause CCM lesions^17,18^. These studies also indicate that alterations not only in the endothelial cells but also in the neurovascular microenvironment may play a role in the formation of CCM lesions^29,30^. To extensively investigate the responsible genes for CCM pathogenesis, whole genome and transcriptome analyses of tissues involving lesions, and lesion micro-environments—other than the focusing solely on *CCM1*, *CCM2*, and *CCM3* gene regions—are of great importance. These findings motivated us to examine genes beyond *CCM1-3* genes. With the great respect for the knowledge of researchers before us, we hypothesized that the gene deregulations observed between patients with hemorrhage and epilepsy may encourage a new perspective to identify the hidden contributors of sporadic CCM pathogenesis.

Interestingly, downregulation of *EGLN1*, *ELAVL4*, *NFE2L2 KCNK18* genes was only detected in epilepsy-related (CvsE) gene list.

EGLN1 catalyzes the alpha protein formation of 4-hydroxyproline in hypoxia-inducible factor (HIF), which is a transcriptional complex that plays a key role in oxygen homeostasis. Analyzing the oncologic signaling pathways that are associated with epilepsy in human glioblastomas (GBMs), a similar study reported that the genes of hypoxia/HIF-1α/STAT5b signaling pathway were decreased in epileptogenic GBMs compared to glioblastomas that do not present with epilepsy^31^. HIF1a is the major transcription factor that provides cellular adaptation to hypoxia^32^. HIF1a expression has been reported to increase in patients with temporal lobe epilepsy^33^. In the cell, EGLN1 functions as a repressor of HIF and when EGLN1 depresses the expression, the expression of the uncontrolled HIF1 will increase.

ELAVL4 is a protein binding protein that regulates mRNA stability, alternative splicing and translation. There are several studies suggesting that *ELAVL* genes are regulating the mechanism of glutamate, which is a major excitatory neurotransmitter, and the expression of several epilepsy related proteins^34,35^.

When the enrichment analysis results are examined, it is noteworthy that the “transcriptional activation by NRF2” obtained from the WikiPathway database (Supp. Fig. S4—CvsE) and the “hypoxia-response via HIF activation” obtained from the Panther database are remarkable for the CvsE gene list (Supp. Fig. S5—CvsE). There are 2 genes (*NFE2L2* and *EGLN1*) related to these features in the CvsE group and there are studies in the literature suggesting that these genes are related to epilepsy. The NRF2 protein, encoded by the *NFE2L2* gene, coordinates the expression of a large number of genes involved in protein signaling, detoxification, antioxidant, anti-inflammatory, and calcium homeostasis. Increased expression of *NFE2L2* gene has been shown to protect the cells against epilepsy by increasing antioxidant properties in neuronal cells^36–38^. As in this study, the decline in the expression of this gene is perhaps a stepping-stone to a series of events leading epilepsy.

*KCNK18* (potassium two pore domain channel subfamily K member 18) gene, which was present in the CvsE group but not in the CvsH group, was categorized under the ‘Tandem pore domain potassium channels’ GO term. *KCNK18* is a potassium channel protein responsible for electrical excitability in the cell. Potassium channels play essential roles in many cellular processes, such as action potential, muscle contraction, hormone secretion, osmotic regulation, and ion flow. Dysfunctions in these channels can cause epilepsy^39^. In a pain model study, it is claimed that the expression of *KCNK18* (TRESK) may decrease and cause neuronal hyperexcitability induced by nerve damage^40^. Another study suggests that TRESK can be a target molecule for the treatment of depression, pain, and epilepsy^41^. Based on this knowledge, downregulated *KCNK18* may cause epilepsy. It is logical to think that CCM patients with loss of function *KCNK18* may develop epilepsy. The possibility of these four promising genes to be candidate genes for epilepsy should be emphasized, and their validation should be ensured by making different analyses.

*USP22*, *EYA1, SIX1*, *OAS3*, *SRMS* genes were significantly downregulated only in the hemorrhage-related (CvsH) gene list.

*USP22* plays key roles in tissue maintenance and tumorigenesis^42^. In Reactome database, USP22 is categorized under “chromatin modifying enzymes” and “chromatin organization” (Supp. Fig. S6—CvsH). Furthermore, *USP22* controls many signaling pathways of vasculature formation and angiogenesis by deubiquitination^43^. Loss of USP22 in mice embryos resulted in failure to establish proper vascular interactions^44^ and knockout of *USP22* caused suppressed angiogenesis and proliferation in lung cancer^45^.

Remarkably, protein–protein interactions demonstrated a direct relation of EYA-1 to SIX1 proteins (Fig. 6b, for more detailed version Supp. Fig. S8), and Enrichment analysis presented that EYA-1 protein is related to “DNA Double Strand Break Response” (Supp. Fig. S6—CvsH). Downregulation of EYA-1 and SIX1 may promote neuronal differentiation and vascular defects^46–48^.

Over-representation Analysis (ORA) categorized OAS3 under “NOD-like receptor signaling pathway” (Supp. Fig. S3—CvsH) which was previously presented as one of the potential new pathways associated with CCM development^49^. *OAS3* was also reported to be upregulated in patients experiencing seizures after spontaneous intracerebral hemorrhage^50^. On the contrary, downregulation of this gene may be a protective way to prevent seizures following intracerebral hemorrhage.

*SRMS* (Src-Related Kinase Lacking C-Terminal Regulatory Tyrosine And N-Terminal Myristylation Sites) gene belongs to the SRC family kinases. Members of this family are functional in several intracellular signaling pathways including proliferation, motility, adhesion, angiogenesis, invasion, and survival. The GO annotation related to *SRMS* gene was identified as ‘Focal Adhesion’ (Supp. Fig. S4—CvsH), but its cellular function is poorly studied.

In a recent study that focused on a specific type of cerebral cavernous angiomas with symptomatic hemorrhage (CASH), the researchers aimed to find biomarkers by comparing CASH patients with Non-CASH patients and healthy individuals via lesional neurovascular unit transcriptome and micro RNAs from blood plasma^20^. When their deregulated gene list of CASH group was compared to our list of genes deregulated in the Hemorrhage group (CvsH DEGs), two common genes were identified: *ACKR2* (Atypical Chemokine Receptor 2) and *MKL2* (MKL/Myocardin-Like Protein 2).

There are several studies related to the vascular function of *ACKR2* and *MKL2*. In a murine study, the increased levels of structural damage, leukocyte infiltration, and fibrotic tissue remodeling markers were observed in the glomerular and tubulointerstitial compartments of Ackr2^−/−^ kidneys, when autologous nephrotoxic nephritis is induced in *Ackr2*-deficient mice. They suggested that *ACKR2* may have an important role in limiting renal inflammation and fibrotic remodeling in progressive nephrotoxic nephritis^51^. Another article reported that *Ackr2*-deficient mice display increased lymphatic vessel density^52^.

*MKL2* is thought to play an essential part in coronary vessel maturation and integrity^53^, and formation of caveolae, which are membrane organelles that play roles in glucose and lipid metabolism and in vascular function^54^. Further studies are required to define the complex role of these genes in CCM disease in further detail.

There are also three commonly identified genes for CvsE and CvsH gene lists (*UCP3, IGHA1, TRAV20).* These genes were downregulated in both lists.

The main function of mitochondrial uncoupling proteins-UCPs such as UCP3 (uncoupling protein 3) is to facilitate the transfer of protons from the outer mitochondrial membrane to the inner mitochondrial membrane by transferring anions from the inner mitochondrial membrane to the outer mitochondrial membrane. UCPs are known to play a role in various processes, such as regulating cellular metabolism and signaling. There are studies showing that UCP2, UCP4 and UCP5 prevent neuronal losses by protecting neurons against the effects of oxidative stress^55^.

In a differential gene expression study conducted with Alzheimer’s patient tissues, it was found that expression of *IGHA1* (immunoglobulin heavy constant alpha 1) increased in hippocampal tissues compared to control group tissues^56^. In another study, it was revealed that the expression of *IGHA1* is increased in epithelium-derived cancer cells^57^. However, there is no article in the literature about the decrease in *IGHA1* expression in brain tissues.

There are several limitations of this study that need to be mentioned. The major challenge was the tissue heterogeneity. The CCM brain tissue samples used in this study were obtained from the lesions containing both veins and the other components of neurovascular units (NVUs), such as neurons, astrocytes or pericytes. Although many genes are cell-specific, we analyzed a combination of different cell types both in patient and control groups. To overcome this limitation, the candidate tissues samples were examined using histological staining, and the samples with high vein-content were chosen. Certainly, for more precise results, using laser-Capture microdissected CCM tissues may be an adequate solution for researchers who have the sufficient infrastructure. Relatedly, a potential follow-up study, based on the recent single-cell gene expression profiling methodology can result in a better resolution. Finally, our sample size was limited because of specific requirement of the samples, such as having no CCM incident in the family, having solely either symptoms of hemorrhage or epilepsy, and having enough quantity of lesional tissue for isolation of total RNA. We suggest these views and limitations are taken into consideration while reading our findings and designing future studies.

Based on the presented results, we propose that distinct deregulated genes might be associated with the development of the CCM, especially in patients with different symptoms. To the best of our knowledge in this context, it is the first report that put forwards novel lists of differentially expressed genes (DEGs) as candidate biomarkers of CCM, based on the transcriptome level analysis in Turkish patients. We believe that these putative CCM-relevant genes need to be experimentally explored in patients with different symptoms in order to ascertain their diagnostic, prognostic and therapeutic potential. Overall, this study provides novel insights into the molecular mechanism underlying the CCM pathology.

## Supplementary Information

Supplementary Information.

## Data Availability

The datasets generated during and/or analyzed during the current study are available from the corresponding author on reasonable request.
