# Supplementary material for: A novel insight into differential expression profiles of sporadic cerebral cavernous malformation patients with different symptoms
Source: Sci Rep. 2021 Sep 29;11:19351. doi: 10.1038/s41598-021-98647-9 (PMC8481309; doi:10.1038/s41598-021-98647-9)

**Supplementary Information**

**A NOVEL INSIGHT INTO DIFFERENTIAL EXPRESSION PROFILES OF SPORADIC CEREBRAL CAVERNOUS MALFORMATION PATIENTS WITH DIFFERENT SYMPTOMS**

Hilal Eren Gozel*^1, 2^; Kivanc Kok^2,3^; Fatma Ozlen^4^; Cihan Isler^4^; Sadrettin Pence^5^

1 Aziz Sancar Institute of Experimental Medicine, Istanbul University, Istanbul, Turkey

2 Regenerative and Restorative Medicine Research Center (REMER), Health Sciences and Technology Research Institute (SABITA), Istanbul Medipol University, Istanbul, Turkey

3 Department of Biostatistics and Medical Informatics, International School of Medicine, Istanbul Medipol University, Istanbul, Turkey

4 Department of Neurosurgery, Cerrahpasa Medical Faculty, Istanbul University-Cerrahpasa, Istanbul, Turkey

5 Department of Physiology, Faculty of Medicine, Istanbul Medeniyet University, Istanbul, Turkey

**Table S1:** Meta table of samples (P: patient; C: control; M: male; F: female; Lesion Location: lesion region from where the sample was obtained).

| Samples | Age | Sex | Symptom | Lesion Location |
| --- | --- | --- | --- | --- |
|  |  |  |  |  |
| P1 | 17 | M | Epilepsy | Bilateral (Multiple) Frontal Lobe |
| P2 | 28 | M | Epilepsy | Left Temporal Lobe |
| P3 | 43 | M | Epilepsy | Left Temporal Lobe |
| P4 | 38 | F | Hemorrhage | Left Frontal Lobe |
| P5 | 74 | F | Hemorrhage | Right Temporal-Insular Region |
| P6 | 52 | F | Hemorrhage | Corpus Callosum |
| C1 | 74 | F | No symptom | Right Parietal Lobe |
| C2 | 45 | F | No symptom | Corpus Callosum |
| C3 | 17 | M | No symptom | Right Frontal Lobe |
| C4 | 36 | M | No symptom | Left Temporal Lobe |
|  |  |  |  |  |

**Table S2:** List of CvsE differentially expressed genes (DEGs). The gene list was obtained by selecting protein-coding genes among top 200 significant differentially expressed probesets from the CvsE comparison. Gene Symbol, Affymetrix probeset IDs, and p value are shown for each DEG.

**Table S3:** List of CvsH differentially expressed genes (DEGs). The gene list was obtained by selecting protein-coding genes among top 200 significant differentially expressed probesets from the CvsH comparison. Gene Symbol, Affymetrix probeset IDs, and p value are shown for each DEG.

**Table S4:** List of CvsEvsH differentially expressed genes (DEGs). The gene list was obtained by selecting protein-coding genes among top 200 significant differentially expressed probesets from the CvsEvsH comparison. Gene Symbol, Affymetrix probeset IDs, and p value are shown for each DEG.

**Fig. S1:** Hierarchical clustering heatmap for the expression of CvsE DEGs. The DGE profile highlights separation of the studied samples into Control (C) and Epileptic (E) groups based on the expression of DEGs. Red color and blue color show relatively higher and lower gene expression, respectively.

**Fig. S2:** Hierarchical clustering heatmap for the expression of CvsH DEGs. The DGE profile highlights separation of the studied samples into Control (C) and Hemorrhagic (H) groups based on the expression of DEGs. Red color and blue color show relatively higher and lower gene expression, respectively.

**Fig. S3:** Over-representation Analysis (ORA), based on the functional annotation information from KEGG database. Results are shown separately for the CvsE, CvsH, and CvsEvsH DEG lists. Significantly enriched KEGG terms (*p*<0.05) are highlighted with asterisks. Top categories (ordered according the p-value in an ascending manner) are shown.

**Fig. S4:** Over-representation Analysis (ORA), based on the functional annotation information from Wikipathway database. Results are shown separately for the CvsE, CvsH, and CvsEvsH DEG lists. Significantly enriched Wikipathway terms (*p*<0.05) are highlighted with asterisks. Top categories (ordered according the p-value in an ascending manner) are shown.

**Fig. S5:** Over-representation Analysis (ORA), based on the functional annotation information from Panther database. Results are shown separately for the CvsE, CvsH, and CvsEvsH DEG lists. Significantly enriched Panther terms (*p*<0.05) are highlighted with asterisks. Top categories (ordered according the p-value in an ascending manner) are shown.

**Fig. S6:** Over-representation Analysis (ORA), based on the functional annotation information from Reactome database. Results are shown separately for the CvsE, CvsH, and CvsEvsH DEG lists. Significantly enriched Reactome terms (*p*<0.05) are highlighted with asterisks. Top categories (ordered according the p-value in an ascending manner) are shown.

**Fig. S7:** Protein-protein interaction network (PPI) analysis for the CvsE DEG list. Nodes represent proteins and edges represent protein-protein interactions. The main interaction map consists of seed proteins (encoded by the DEGs) and their direct interaction partners (first degree interactors identified by the String database). The DEGs without any connection are listed at the bottom of the figure.
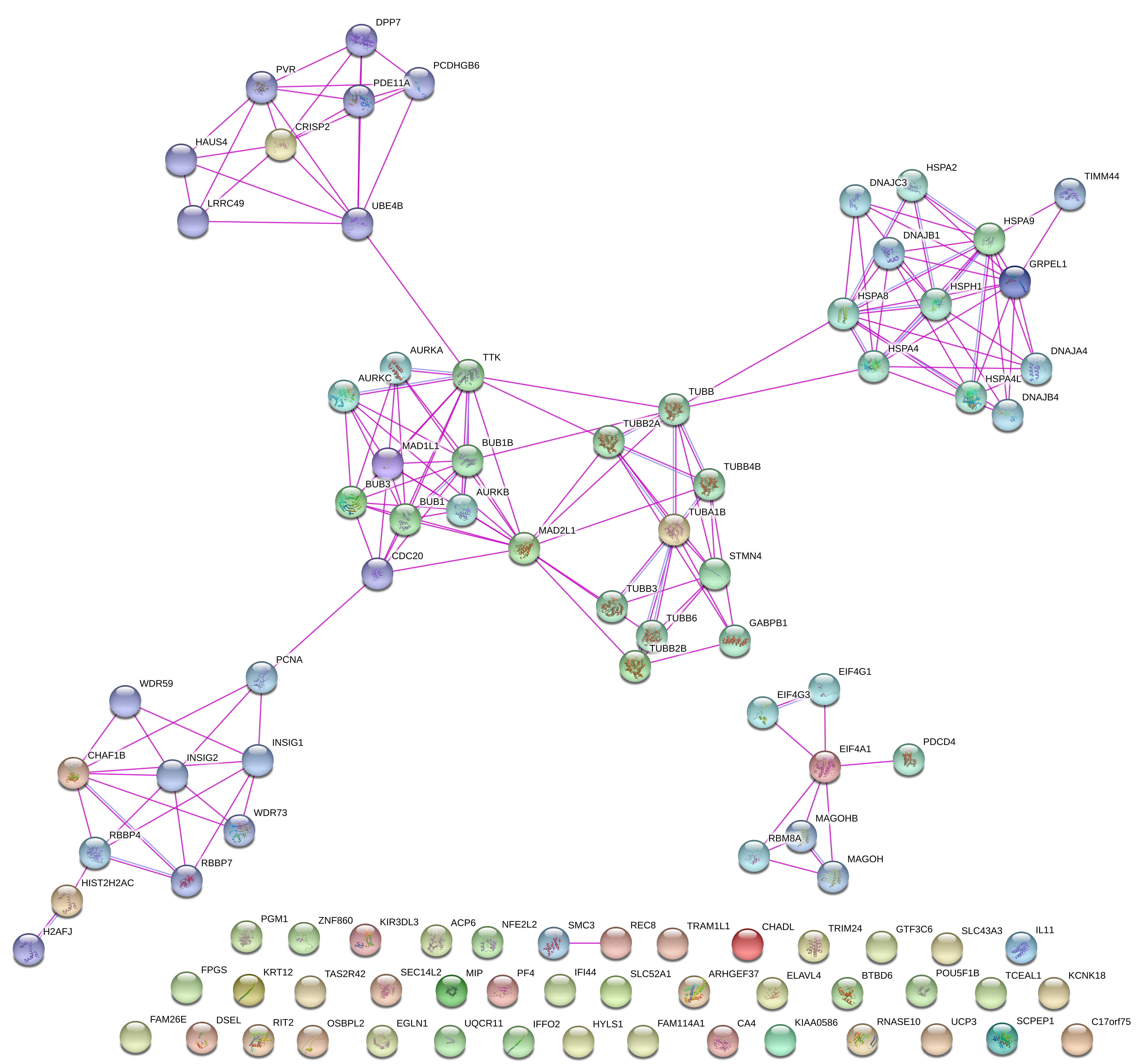


**Fig. S8:** Protein-protein interaction network (PPI) analysis for the CvsH DEG list. Nodes represent proteins and edges represent protein-protein interactions. The main interaction map consists of seed proteins (encoded by the DEGs) and their direct interaction partners (first degree interactors identified by the String database). The DEGs without any connection are listed at the bottom of the figure.
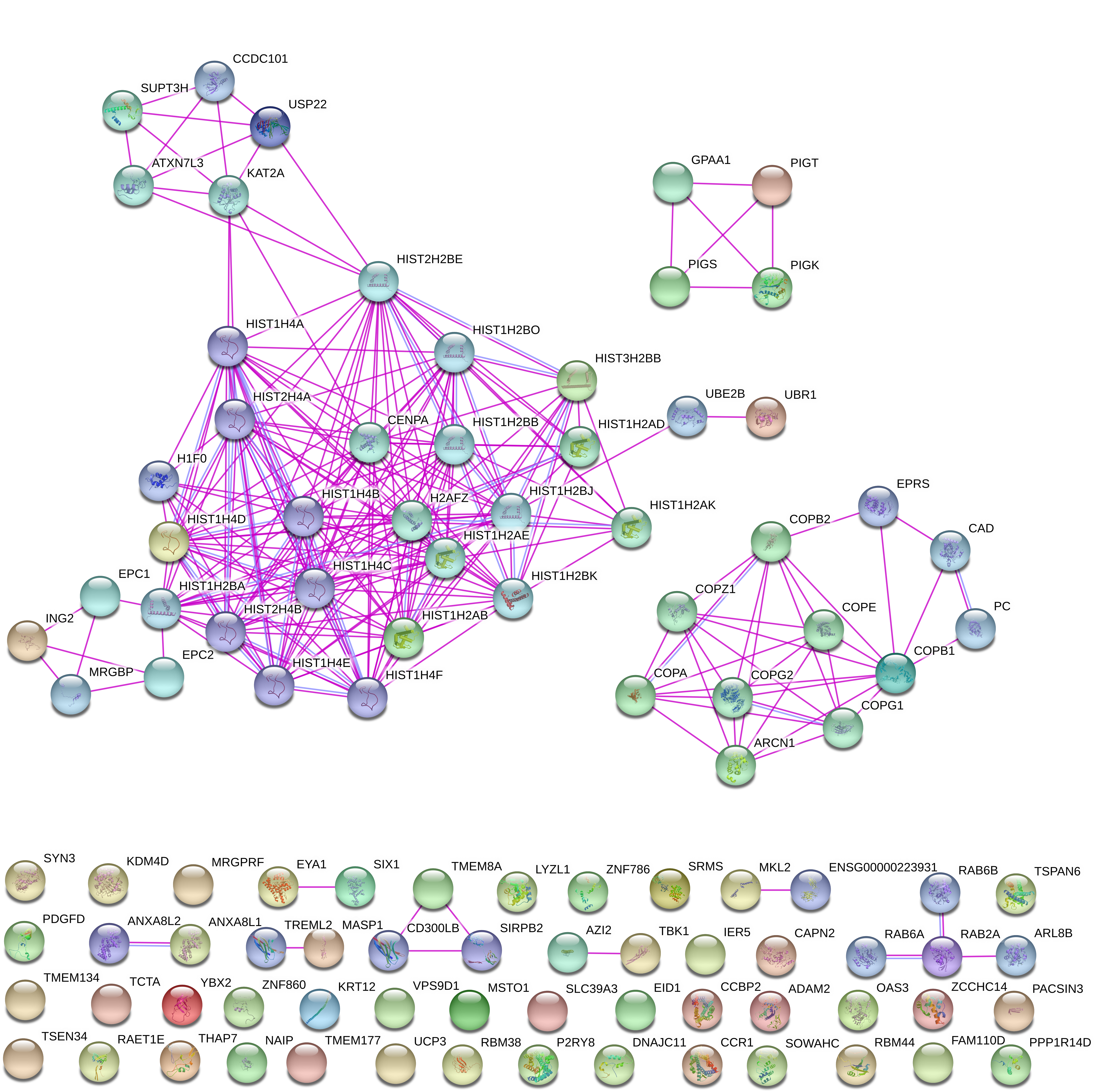


**Fig. S9:** Protein-protein interaction network (PPI) analysis for the CvsEvsH DEG list. Nodes represent proteins and edges represent protein-protein interactions. The main interaction map consists of seed proteins (encoded by the DEGs) and their direct interaction partners (first degree interactors identified by the String database). The DEGs without any connection are listed at the bottom of the figure.


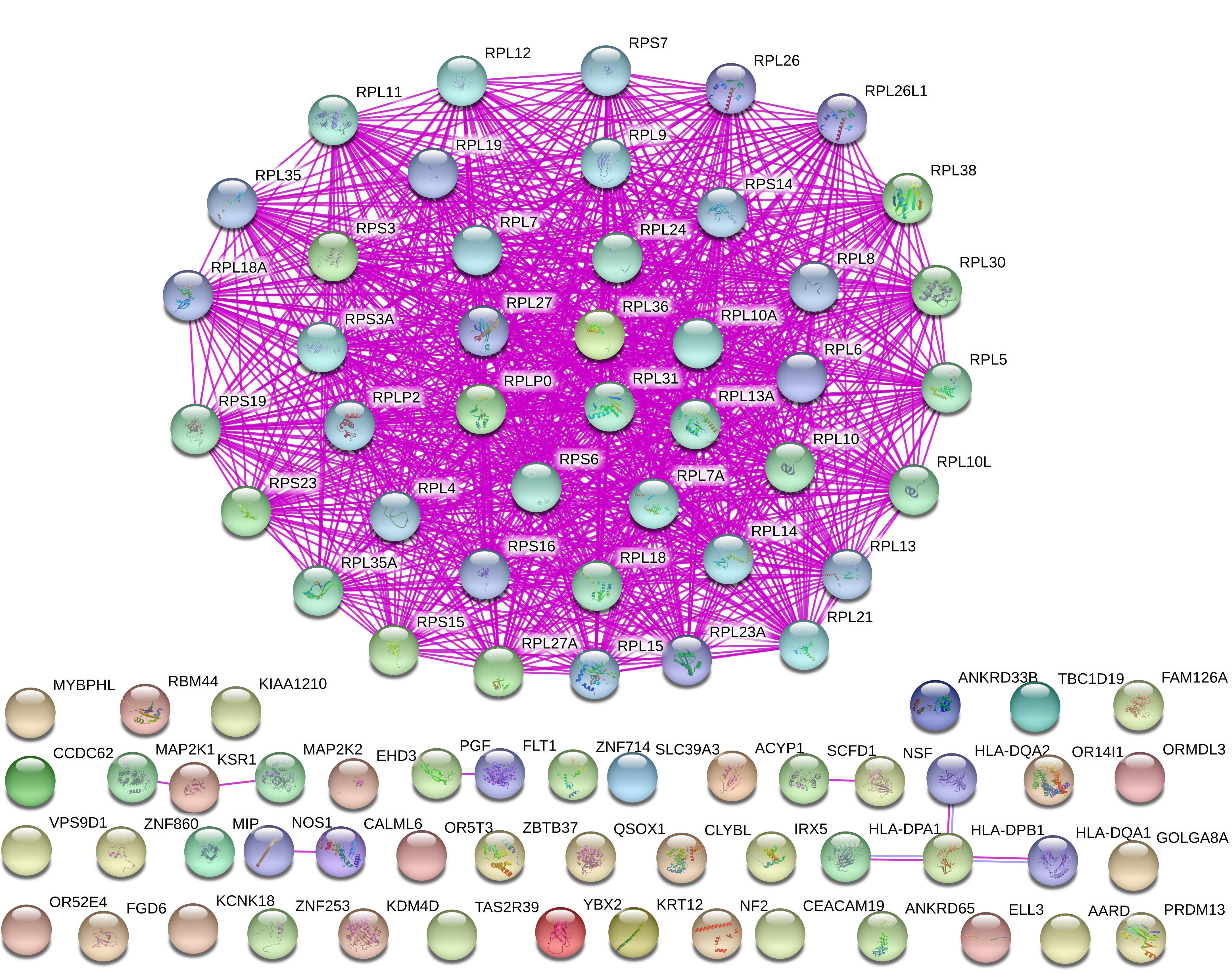

Supplement: Supplementary file 1 — Supplementary Information. [file 41598_2021_98647_MOESM1_ESM.docx]
